# Reply to Cetkovic et al. Comment on “Meneghini et al. The Impact of Nutritional Therapy in the Management of Overweight/Obese PCOS Patient Candidates for IVF. *Nutrients* 2023, *15*, 4444”

**DOI:** 10.3390/nu16030439

**Published:** 2024-02-01

**Authors:** Caterina Meneghini, Claudia Bianco, Francesco Galanti, Valentina Tamburelli, Alessandro Dal Lago, Emanuele Licata, Mariagrazia Gallo, Cristina Fabiani, Roberta Corno, Donatella Miriello, Rocco Rago

**Affiliations:** 1 Physiopathology of Reproduction and Andrology Unit, Sandro Pertini Hospital, Via dei Monti Tiburtini 385/389, 00157 Rome, Italy; 2 Department of Science, University “Roma Tre”, 00146 Rome, Italy; 3 Independent Researcher, 00100 Rome, Italy

Thanks for your comment [1]. In our work, we have stated that the Mediterranean diet is an indisputable cornerstone of nutrition which certainly leads to an improvement in the patient’s overall state of health [2]. However, our objective was to demonstrate that a VLCKD represents a valid alternative that allows patients to achieve their goals in a shorter period (120 days) and then proceed to medically assisted procreation (PMA). Furthermore, after the intensive phase, our patients reintegrate a low-calorie Mediterranean diet during pregnancy. This further demonstrates that we hold the Mediterranean diet in high regard.

We are continuing to recruit patients, and therefore our sample is destined to become larger.

We thank you for the attention that has been paid to our work and for your valuable suggestions.
